# Intrinsically Charged Peptide Nanomembranes as Self-Organized Electrostatic Interfaces in Confined Ionic Liquids

**DOI:** 10.1021/acs.langmuir.6c03513

**Published:** 2026-09-16

**Authors:** Karinna Mendanha, Tertius L. Fonseca, Guilherme Colherinhas

**Affiliations:** † Departamento de Física, CEPAE, 176873 Universidade Federal de Goiás, 74690-900 Goiânia, Goiás, Brazil; ‡ Instituto de Física, Universidade Federal de Goiás, 74690-900 Goiânia, Goiás, Brazil

## Abstract

Self-assembled biomolecular materials provide a promising route for designing soft, sustainable, and molecularly tunable electrostatic interfaces that are relevant to energy storage, iontronics, and bioinspired electrochemical systems. However, the molecular mechanisms by which charged peptide assemblies organize confined ionic environments and generate interfacial electrostatic gradients remain poorly understood. Here, we use fully atomistic molecular dynamics simulations to investigate self-assembled peptide nanomembranes composed of the bolaamphiphilic sequences EFL_4_FE and RFL_4_FR immersed in the ionic liquid [PYR]­[TFSI]. We examine these nanosheets as intrinsically charged supramolecular interfaces, whose surface charges arise from peptide composition, predefined protonation states, and supramolecular organization. The distinct charge characteristics of the two peptide sequences produce sequence-dependent ionic organization with preferential recruitment of cations or anions according to the local sign of the peptide charge. In both systems, localized peptide charges induce compact counterion accumulation, co-ion depletion, charge density oscillations, and electrical double-layer (EDL)-like organization. Consequently, the confined ionic liquid exhibits pronounced interfacial structuring and electrostatic potential gradients governed by the spatial distribution of charges within the peptide nanomembranes. Moreover, the electrostatic potential profiles indicate that the interfacial perturbation extends into the supramolecular membrane rather than being restricted to the electrolyte-facing surface. Within the limitations of the nonreactive classical fixed-charge model, these results provide molecular-level evidence that intrinsically charged peptide nanomembranes can organize confined ionic liquids and sustain electrostatic gradients across supramolecular interfaces. Their potential use as electrostatic transduction elements or components of iontronic and electrochemical devices requires further investigation, explicitly addressing electronic transport, external electrical coupling, and device-level operation.

## Graphical abstract

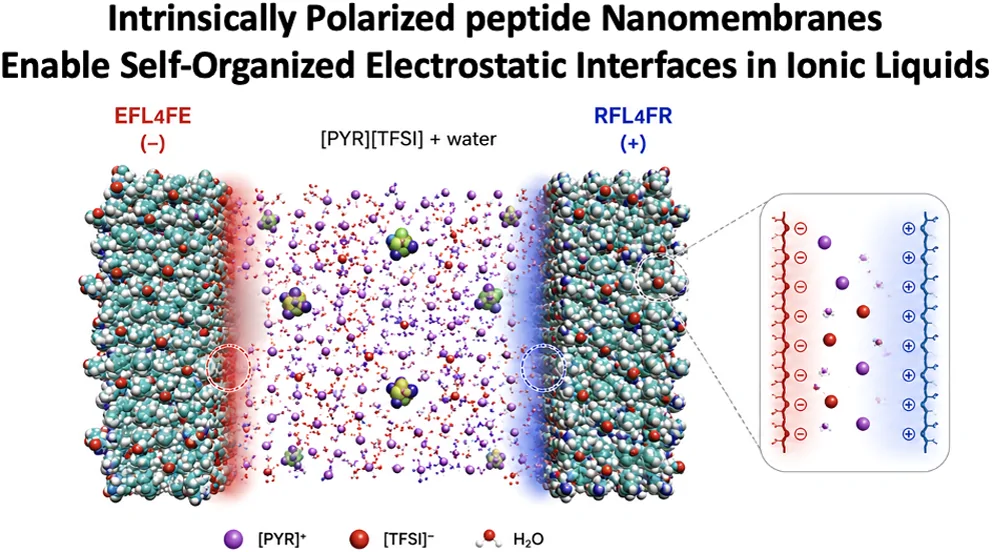

## Introduction

The development of sustainable and molecularly tunable electrostatic materials is increasingly relevant to energy storage, wearable electronics, bioelectronics, and autonomous systems. Organic and bioinspired materials are attractive because they may provide lightweight, flexible, less toxic, and potentially biodegradable interfaces. Beyond their use in electrochemical devices, these systems offer model platforms for investigating how molecular structure and interfacial chemistry govern charge separation, ionic organization, and EDL formation.^1^ Organic electrochemical materials nevertheless continue to face limitations related to conductivity, structural stability, and long-term performance.^1^ Strategies based on redox-active molecules and pseudocapacitive mechanisms,^2^ as well as conductive additives such as amorphous carbon,^3^ have been widely explored to address these limitations. However, these approaches are based on externally polarized conductive frameworks, whereas less attention has been devoted to molecular systems in which electrostatic interfaces emerge intrinsically from the supramolecular organization. In this context, the key question is not only whether biomolecular assemblies can function as electrode components but also whether their intrinsic molecular charge can generate and sustain organized electrostatic interfaces in confined ionic-liquid environments.

Self-assembling peptides provide a particularly suitable platform for addressing this question because they form highly organized interfaces whose structural and electrostatic properties can be governed by the peptide sequence, predefined protonation states, hydrogen bonding, hydrophobic packing, and interfacial charge distribution. Such features make peptide-based assemblies attractive not only for sustainable energy-related devices but also as model systems for studying how biomolecular self-assembly controls electrolyte organization and interfacial electrostatics.^4^ In addition, peptides and amino acid-based materials offer biocompatibility, biodegradability, and chemical tunability, reinforcing their relevance as sustainable alternatives to conventional inorganic materials.^5^

The morphology and surface chemistry of peptide assemblies strongly influence their interfacial electrostatic behavior. Nanotubes and nanosheets can provide large accessible surface areas and extensive contact with electrolytes, thereby favoring interfacial ionic organization.^6^ The peptide–electrolyte interface is therefore the central region for understanding the molecular processes examined in these systems. Electrostatic interactions, hydrogen-bonding networks, wettability, surface roughness, molecular packing, and confinement can collectively govern ion adsorption, counterion enrichment, co-ion depletion, and charge density oscillations near charged peptide surfaces.^7^ Therefore, understanding these phenomena consequently requires moving beyond a purely device-centered description and focusing on the molecular mechanisms by which peptide interfaces organize surrounding ionic species.

Hybrid organic–inorganic systems have also been widely investigated as a way to combine molecular functionality with improved conductivity, mechanical stability, and structural durability. Organic–inorganic architectures can introduce electrochemically active sites and enhance charge transport pathways,^8^ while inorganic coatings may increase the stability of peptide-based electrodes.^9^ Similarly, graphene/organic composites exploit the high surface area and conductivity of graphene together with the redox or interfacial activity of organic components.^10^ Although these hybrid strategies remain important for practical energy storage applications, they also demonstrate that interfacial behavior cannot be understood solely in terms of bulk conductivity. The molecular structure, local surface charge, electrolyte accessibility, and ionic organization must also be considered. Isolating these molecular contributions is therefore necessary for determining whether biomolecular assemblies can generate organized electrostatic interfaces independent of conductive supports or externally imposed electrode polarization.

Ionic liquids are particularly relevant for investigating interfacial electrostatics because they combine high ionic density and thermal stability with complex molecular organization near charged surfaces.^11^ Unlike dilute electrolytes, ionic liquids often form compact, oscillatory, and strongly correlated layers governed by ion packing, Coulombic interactions, overscreening, and confinement effects. Their organization is therefore highly sensitive to the sign, magnitude, and spatial heterogeneity of the surface charges. Ionic liquids consequently provide an appropriate environment for probing how intrinsically charged biomolecular interfaces structure dense ionic media in the absence of an externally applied electrode polarization. Amino acid-based ionic liquids further broaden this field by incorporating biocompatibility and potential sustainability into the design of electrochemical and interfacial systems.^11^

Interfacial stability, wettability, hydrogen-bond reorganization, and self-regenerating behavior are important considerations in the development of sustainable and long-lived electrochemical systems.^12^ Previous investigation of biodegradable peptide/ionic liquid models further showed that strong interfacial interactions and high peptide surface charge densities affect capacitance-like responses and ionic organization.^7^ These results support treating peptide assemblies not only as possible electrode components but also as intrinsically charged supramolecular interfaces capable of directing electrolyte organization. However, it remains unclear how peptide nanomembranes with distinct charge characteristics control the identity and spatial distribution of interfacial ions, the formation of charge density oscillations, and the resulting electrostatic potential profiles. Clarifying these relationships is necessary to distinguish equilibrium biomolecular interfacial electrostatics from the operational response of an externally polarized electrochemical device.

Thus, in this context, peptide nanomembranes stand out because they can self-assemble into highly organized and functionally active supramolecular architectures in which molecular packing, surface charge, and electrolyte accessibility are intrinsically coupled.^13^ Unlike conventional carbon-based electrodes, whose interfacial charge is usually imposed externally, peptide nanomembranes may sustain localized surface charges arising directly from their molecular composition, predefined protonation states, and supramolecular arrangement. These charges may induce equilibrium ionic structuring in the absence of externally imposed electrode polarization. Hereafter, the term “EDL-like organization” refers specifically to the combined occurrence of counterion enrichment, co-ion depletion, interfacial ionic layering, and charge density oscillations near the intrinsically charged peptide surfaces. The qualifier “like” is retained because the present fixed-charge simulations employed here do not represent an externally polarized or operational electrochemical device. In this work, we perform fully atomistic molecular dynamics simulations of intrinsically charged EFL_4_FE and RFL_4_FR peptide nanomembranes immersed in the ionic liquid [PYR]­[TFSI]. Rather than treating these systems primarily as conventional supercapacitor electrodes, we investigate them as self-organized biomolecular electrostatic interfaces. Specifically, we determine how the distinct charge characteristics of the two peptide sequences influence ionic-liquid layering, EDL-like organization, charge density distributions, electrostatic potential profiles, and the spatial propagation of interfacial electrostatic perturbations. This approach preserves the relevance of peptide nanomembranes to energy-related materials while placing the main contribution within the broader framework of biomolecular interfacial electrostatics.

## Methodology

### Peptide Nanomembrane Construction

The construction of the peptide nanomembranes employed in this work was based on the atomistic protocol previously described for the bolaamphiphilic EFL_4_FE and RFL_4_FR systems, originally developed for the structural and energetic investigation of self-assembled peptide nanosheets.^14^ Both sequences are composed of a hydrophobic core formed by four consecutive leucine residues (L_4_), terminally functionalized with aromatic phenylalanine (F) residues and charged glutamic acid (E) or arginine (R) groups. Consequently, the EFL_4_FE and RFL_4_FR sequences exhibit a bolaamphiphilic character, in which charged hydrophilic terminations are separated by a highly organized hydrophobic central domain. Under the simulated alkaline conditions employed to stabilize the membrane structures, the terminal groups are represented in charged states, found in the COO^–^ and NH^3+^ forms, leading to distinct net surface charges on the membranes. Thus, the EFL_4_FE nanosheet presents one predominantly negatively charged surface, whereas the RFL_4_FR nanosheet exhibits one predominantly positively charged surface. This intrinsic charge asymmetry constitutes the molecular basis for the formation of the confined biomolecular electrostatic interface investigated in this work. The initial membrane structures were constructed in an antiparallel β-sheet conformation, as previously reported for these systems, preserving the high degree of intermolecular packing associated with π–π interactions between aromatic residues and the extensive hydrogen-bonding network among the peptides.^14^

The membranes were modeled through the regular juxtaposition of peptides in a two-dimensional periodic network, forming self-assembled monolayers. For each system, 64 peptides were organized into a continuous planar structure, reproducing an infinite nanosheet by means of periodic boundary conditions. The structural arrangement directly followed the model described for the original EFL_4_FE and RFL_4_FR nanosheets, in which lateral hydrophobic interactions promote high structural compactness, while the orthogonal direction is stabilized by interchain hydrogen bonds.^14^ The high supramolecular organization of these structures results in rigid and stable membranes, exhibiting thicknesses compatible with experimental SAXS values previously reported for these systems.^14^ The selection of these systems is mainly associated with their ability to sustain high and spatially organized surface charge densities, which are central for investigating spontaneous ionic-liquid structuring and EDL-like organization at biomolecular interfaces. Importantly, the surface charge density was not introduced as an independently adjustable electrode parameter. Instead, an effective surface charge density was calculated as (σ = *Q*/*A*), where *Q* is the sum of the formal charges of the solvent-exposed peptide groups in their predefined protonation states and *A* is the average projected interfacial area of the assembled nanosheet after equilibration. Thus, the values reported in Table 1 arise from the peptide composition, predefined protonation states, and equilibrium lateral packing of the EFL_4_FE and RFL_4_FR assemblies, rather than from an externally imposed uniform charge density.

**Table 1:** Composition and Simulation-Cell Dimensions of the Peptide Nanomembrane and Confined Peptide/Electrolyte Interfacial Systems Investigated in This Work^a^

Information	EFL_4_FE in water	RFL_4_FR in water	EFL_4_FE In [PYR][TFSI]	RFL_4_FR In [PYR][TFSI]	Confined model
# peptides EFL_4_FE	64	--	64	--	64
# peptides RFL_4_FR	--	64	--	64	64
# Cl^–^ ions	--	128	--	128	--
# K^+^ ions	128	--	128	--	--
# [PYR][TFSI] pair	--	--	480	480	900
# water molecules	9993	9871	--	--	45
Final *x*-axis [nm]	9.07	9.29	8.64	8.80	8.80
Final *y*-axis [nm]	4.62	4.73	4.40	4.49	4.49
Final *z*-axis [nm]	9.17	8.89	8.87	8.79	50.00
Final σ [e/nm^2^]	–3.06	+2.91	–3.37	+3.24	±3.24

a The table reports the number of EFL_4_FE and RFL_4_FR peptides, charge-neutralizing ions, [PYR]­[TFSI] ion pairs, and water molecules used in each simulated system, including isolated peptide membranes in water, isolated peptide membranes in [PYR]­[TFSI], and the assembled confined interfacial model. Final box dimensions along the *x*, *y*, and *z* axes are also provided to describe each system. The final surface charge density, σ, is reported for the charged membrane surface, with negative values assigned to EFL_4_FE, positive values assigned to RFL_4_FR, and ±σ used for the assembled confined peptide/electrolyte model.

Additional information regarding the spatial charge distribution across the membrane interfaces is provided in Figure S1 of the Supporting Information, which presents the charge density profiles along the *z*-direction for the peptide nanomembranes in ionic liquid, further highlighting the strong electrostatic asymmetry induced by the oppositely charged membranes.

### Force Fields and Simulation Protocol

All molecular dynamics simulations (MD) were performed using the GROMACS package,^15,16^ maintaining methodological compatibility with previously published studies involving peptide nanosheets and biodegradable electrochemical systems.^7^ The CHARMM36 force field^17–19^ was employed to describe the peptide structures, including the refined CMAP potentials for peptide backbone conformational sampling. The TIP3P model^20–22^ was used for explicit water representation in aqueous simulations. For the ionic liquid [PYR]­[TFSI], 1-butyl-1-methylpyrrolidinium-bis­(trifluoromethylsulfonyl)­imide, the parameters were adapted from classical CHARMM-compatible formalisms,^22,23^ preserving an adequate description of Coulombic and dispersive interactions as well as the interfacial organization of the electrolyte. Long-range electrostatic interactions were treated using the Particle Mesh Ewald (PME) method,^23^ whereas van der Waals interactions were described through Lennard–Jones potentials with a cutoff distance of 1.2 nm. All bonds involving hydrogen atoms were constrained using the LINCS algorithm,^24^ allowing the use of a 2 fs integration time step. Temperature control was performed using the velocity-rescaling thermostat,^25^ while pressure, when employed in NPT simulations, was controlled through the semi-isotropic Parrinello–Rahman barostat,^26^ maintaining a temperature of 300 K and a pressure of 1 atm.

Initially, the EFL_4_FE and RFL_4_FR membranes were individually simulated in an aqueous environment in order to obtain structurally stable membrane conformations. Each system was inserted into periodic simulation boxes containing sufficient explicit water to fully solvate both membrane surfaces. Following initial construction, the systems were subjected to energy minimization using the steepest descent algorithm until convergence of maximum forces below 10^3^ kJ mol^–1^ nm^–1^ was achieved. Subsequently, sequential equilibration protocols involving 5 ns in the NVT ensemble followed by 5 ns in the NPT ensemble were alternately performed until structural relaxation and stabilization of system density and potential energy were obtained. Production simulations were then carried out for 100 ns in the NPT ensemble, and 10^5^ configurations were saved for analysis.

Subsequently, the same membranes were individually simulated in the ionic liquid [PYR]­[TFSI], where each membrane surface was placed in direct contact with an electrolyte. This stage aimed primarily to investigate electrolyte reorganization at the membrane/electrolyte interfaces and to evaluate the effects of the intrinsic peptide surface charge distribution on ionic-liquid layering over the distinct nanomembrane surfaces. In analogy with previously observed behavior in amino acid ionic liquid systems,^27^ it was expected that the charged nanosheet surfaces would induce strong spatial reorganization of electrolyte ions, promoting counterion accumulation near the charged membrane regions. For each system, successive minimization, pre-equilibration, and production stages were performed following the same thermodynamic protocol employed in aqueous simulations. In both stages, for water-solvated and ionic liquid-solvated membranes, counterions were added to ensure overall charge neutrality of the systems, which was necessary because the nanomembranes possess electrically charged surfaces.

Table 1 summarizes the number of particles employed in each simulation. During these initial simulations involving individually solvated peptide nanomembranes, structural parameters such as mass density profiles, conformational stability, membrane thickness, water or ionic-liquid distributions at the interfaces, and hydrogen-bond organization were monitored. Particular attention was devoted to the penetration of water molecules into the internal regions of the nanosheets, since previous studies^28–31^ demonstrated that water molecules can occupy spaces between peptide side chains, partially stabilizing the supramolecular structure through hydrophilic interactions, as well as to the presence of well-defined ionic layers at the nanomembrane surface when immersed in the ionic liquid. These results were used as reference systems for comparison with the confined peptide/electrolyte interfacial model.

### Confined Peptide/Electrolyte Model

After stabilized nanosheet configurations were obtained in [PYR]­[TFSI], the confined peptide/electrolyte interfacial model was constructed. The system was assembled using one EFL_4_FE nanosheet and one RFL_4_FR nanosheet positioned parallel to each other along the *z*-direction of the simulation box. The charged surfaces of the membranes were oriented toward the internal region containing the confined ionic liquid, while the opposite surfaces remained oriented toward the external region of the system. This configuration was inspired by previous peptide-based electrochemical models^7^ but is interpreted here primarily as a biomolecular electrostatic interface rather than as a conventional externally polarized supercapacitor device. The membranes were separated by approximately 12 nm between their charged surfaces, allowing the formation of a confined electrolyte region suitable for investigating spontaneous ionic liquid organization and EDL-like formation. The electrolyte consisted of 900 ionic pairs of [PYR]­[TFSI] and 45 water molecules (see Table 1), which was sufficient to maintain residual hydration without significantly compromising either the ionic-liquid organization or the nanomembrane structure. An important aspect concerns the dimensions of the nanomembranes, since both present very similar total surface areas for the same number of composing peptides, favoring an electrically balanced interfacial model within a rectangular box defined by the nanomembrane transverse area and ensuring comparable intrinsic surface charge densities, expressed in e/nm^2^, for the two oppositely charged peptide interfaces.

The confined peptide/electrolyte interfacial model was initially subjected to energy minimization using the steepest descent algorithm, followed by successive equilibration stages in the NVT ensemble to ensure correct system confinement. The complete system was additionally isolated within the simulation box and positioned at the center of a box with *z* = 50 nm, thereby creating a vacuum region to reduce undesired interactions between periodic images of the nanomembranes along this direction,^7,32–35^ while maintaining *xy*-plane periodicity and characterizing an effectively extended interfacial system. During pre-equilibration, soft positional restraints were temporarily applied to peptide heavy atoms to prevent abrupt structural distortions arising from the initial electrolyte reorganization. After stabilization of system density and temperature, these restraints were completely removed, and all peptide atoms remained fully flexible throughout the 100 ns production simulation. Thus, the membrane conformations, local surface fluctuations, and peptide–electrolyte interactions analyzed in this work evolved without positional constraints during the production stage. The final confined peptide/electrolyte model was simulated for 100 ns under the NVT ensemble during the production stage. This trajectory was used to analyze EDL-like organization, spatial charge distribution, ion organization near the peptide interfaces, and electrostatic potential profiles across the confined interfacial model. In analogy with previous studies involving biodegradable peptide nanosheet systems, capacitance-like electrostatic descriptors were estimated from the spatial charge distribution and electrostatic potential profile obtained through numerical integration of the one-dimensional Poisson equation using the GROMACS implementation.^7,15,16^ These quantities are interpreted here as descriptors of interfacial electrostatic response rather than as direct operational metrics of a conventional supercapacitor device. The present simulations were performed using a classical nonreactive fixed-charge force field. Consequently, the molecular charge distribution remains fixed throughout the trajectory, and processes such as explicit electronic polarization, proton transfer, bond breaking or formation, electron transfer, electrolyte decomposition, and faradaic reactions are not represented. The calculated structural and electrostatic properties should therefore be interpreted within the scope of this classical molecular description.

### Structural and Electrostatic Analyses

Structural and electrostatic analyses involved calculations of mass density profiles, charge density distributions, and electrostatic potentials along the direction perpendicular to the membranes. EDL-like organization was investigated particularly at the peptide/electrolyte interfaces, separately considering the behavior of the positive and negative peptide surfaces. To evaluate the statistical stability of the electrostatic observables, the production trajectory was divided into 5 independent temporal blocks of 20 ns each. Charge density profiles and the corresponding electrostatic potential profiles were calculated independently for each block. The values reported for Φ^–^, Φ^+^, and ΔΦ correspond to the mean over these blocks, and the associated uncertainties represent the standard deviation among the block-averaged values. The ion distribution near the nanosheets was used to evaluate the degree of counterion accumulation and the possible occurrence of ionic semipermeation on peptide surfaces, a phenomenon previously associated with enhanced interfacial electrostatic response in biodegradable peptide-based systems.^7^ Finally, the obtained results were used to directly correlate supramolecular structure, interfacial electrolyte organization, and electrostatic response, allowing an atomistic evaluation of peptide nanomembrane behavior in confined ionic-liquid electrostatic interfaces. For conversion of the formal electrostatic energy per unit area into an energy-like volumetric descriptor, an effective interfacial thickness of 10 nm, corresponding to the distance between the opposing membrane surfaces, was employed. Figures 1 and 2 present the representation of all systems simulated in this work. For all systems, visual analyses were performed using VMD software,^36^ and the initial simulation boxes were assembled using PACKMOL software.^37^ Further analyses were performed using the tools available in the GROMACS package,^15,16,38^ and graphs were created using Python programming.

**Figure 1:**
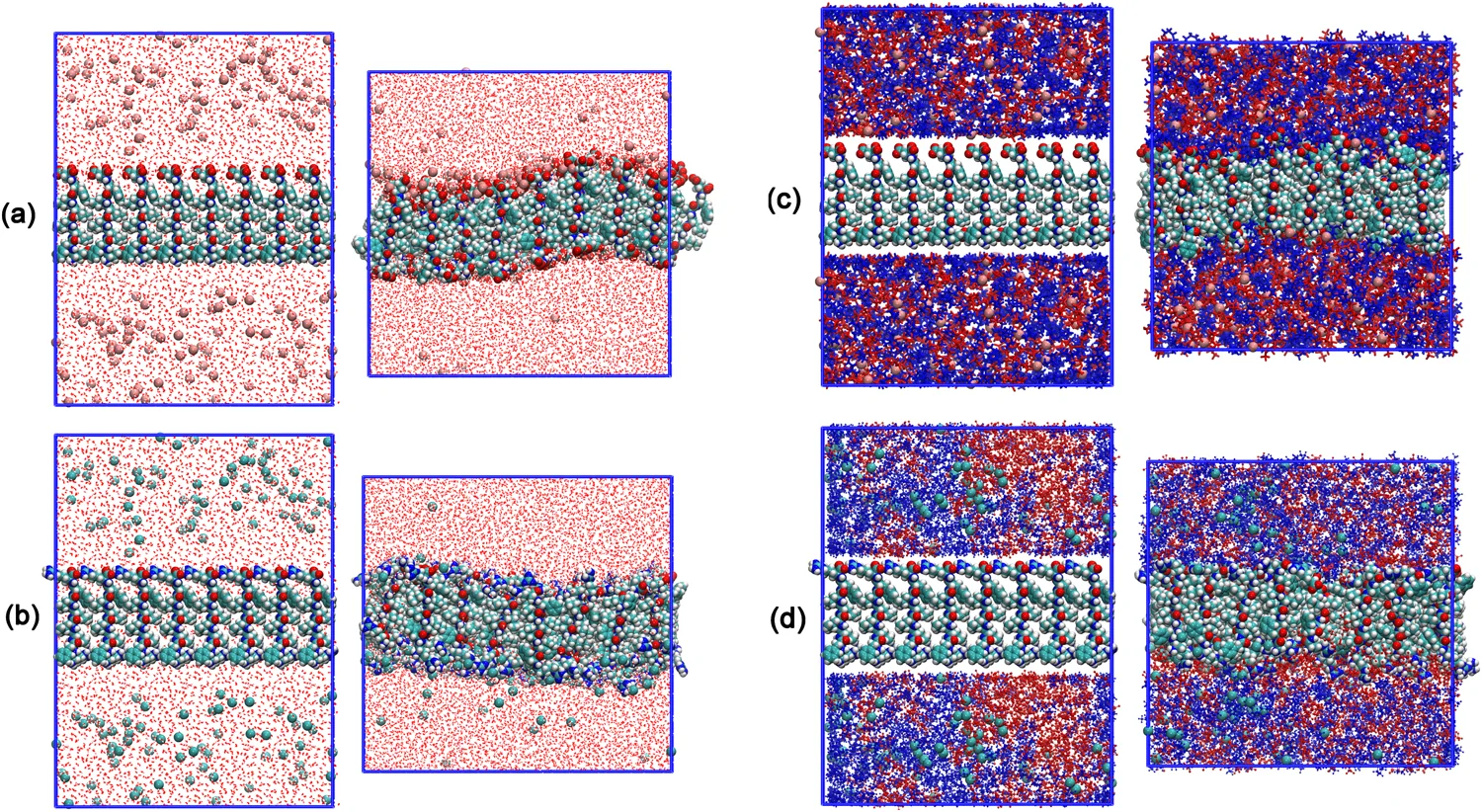
Initial (left) and final (right) configurations of the isolated EFL_4_FE (a, c) and RFL_4_FR (b, d) peptide nanomembranes simulated in water (a, b) and [PYR]­[TFSI] (c, d). K^+^ and Cl^–^ counterions are represented as spheres, [PYR]^+^ in blue, [TFSI]^−^ in red, and water as red/white lines. Peptides are shown as van der Waals spheres (O, red; C, cyan; H, white; N, blue).

**Figure 2:**
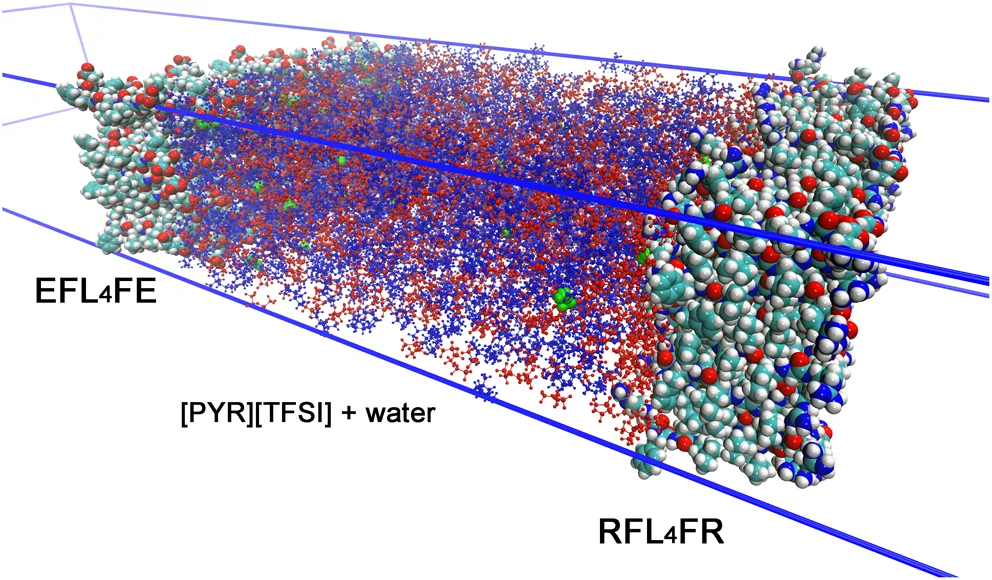
Final atomistic configuration of the confined EFL_4_FE/[PYR]­[TFSI]/RFL_4_FR interfacial model. The oppositely charged peptide surfaces face the confined ionic-liquid region. Peptides are represented as van der Waals spheres (O, red; C, cyan; H, white; N, blue), with [PYR]^+^ in blue and [TFSI]^−^ in red. Residual water and counterions are omitted in selected regions for clarity.

## Results and Discussion

The results are presented by progressively connecting the structural stability of the peptide nanomembranes, the spontaneous organization of the confined ionic liquid, and the resulting electrostatic response at the peptide/electrolyte interface. This organization allows the system to be analyzed not as a conventional externally polarized supercapacitor but as a self-organized biomolecular electrostatic interface in which localized peptide surface charges drive ion layering, counterion accumulation, and EDL-like formation. Accordingly, the following sections first examine the structural integrity and interfacial geometry of the EFL_4_FE and RFL_4_FR nanomembranes under ionic liquid confinement, then analyze the density profiles and ionic layering of the confined [PYR]­[TFSI] electrolyte, and finally discuss how this interfacial organization gives rise to electrostatic potential gradients and capacitance-like descriptors.

### Structural Stability of Peptide Nanomembranes in Confined Ionic Liquid

Figure 3 presents the structural configuration of the confined peptide/electrolyte interfacial model after stabilization by molecular dynamics simulations, highlighting the spatial arrangement of the ionic liquid [PYR]­[TFSI] confined between the EFL_4_FE and RFL_4_FR peptide nanomembranes. The system was constructed by using two peptide nanosheets with opposite intrinsic surface charges facing each other, creating a well-defined confined region suitable for the emergence of interfacial electrostatic organization. The EFL_4_FE and RFL_4_FR nanosheets remain arranged approximately parallel to each other along the *z*-direction, delimiting the ionic-liquid domain and preserving the intended interfacial geometry throughout the simulation. This configuration provides the structural basis for analyzing how intrinsically charged peptide surfaces influence the organization of confined electrolytes.

**Figure 3:**
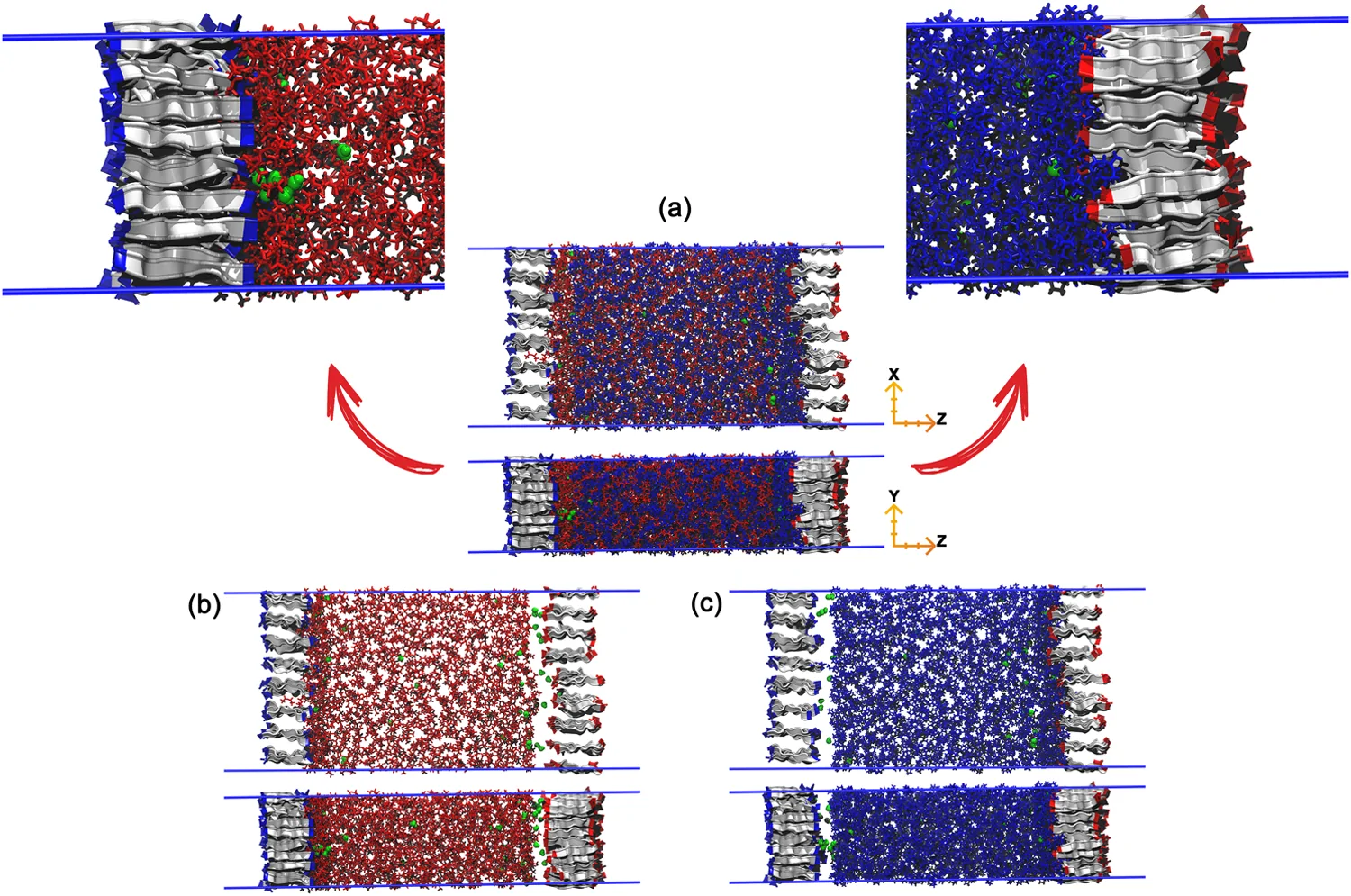
Structural organization of the confined peptide/electrolyte model after equilibration. (a) Orthogonal views of the EFL_4_FE and RFL_4_FR nanomembranes separated by [PYR]­[TFSI]. Enlarged views show the positively charged RFL_4_FR interface (b) and negatively charged EFL_4_FE interface (c). Peptides are shown in gray, charged terminal groups in red and blue, [PYR]^+^ in blue, [TFSI]^−^ in red, and water in green.

The final configuration shown in Figure 3(a) indicates that both peptide nanomembranes preserve their supramolecular integrity under ionic-liquid confinement. The absence of large-scale disruption is supported by the preservation of the membrane density profiles and the maintenance of the intermembrane separation during the production trajectory, suggesting that the peptide assemblies remain structurally stable in contact with the dense ionic medium. The hydrophobic peptide domains maintain a compact organization, while the charged terminal groups remain exposed to the confined electrolyte region. This preservation of the nanosheet architecture is essential, because the electrostatic behavior investigated in the following sections depends directly on the maintenance of spatially organized peptide surface charges. Importantly, the structural stability observed here does not result from artificial rigidity during the production stage since all positional restraints were removed before the 100 ns production simulation. The peptide nanomembranes therefore retained their local conformational flexibility and underwent thermally driven fluctuations while preserving their overall supramolecular organization. Such fluctuations are expected to modulate the instantaneous distances between charged peptide groups and electrolyte ions, thereby influencing the local ion orientation, packing, hydrogen-bonding patterns, and transient counterion accumulation. They may also introduce short-time variations in the local charge density and electrostatic potential profiles. However, because the membranes maintain their global architecture, interfacial orientation, and spatially organized surface charge distributions throughout the trajectory, these local fluctuations are not expected to alter the qualitative formation of the double-layer-like structures. Instead, the density and electrostatic potential profiles reported here represent trajectory-averaged responses that incorporate the configurational fluctuations of fully flexible peptide interfaces.

The detailed views in Figure 3b,c further show that the peptide/electrolyte boundaries remain well-defined after molecular dynamics stabilization. The charged surfaces of the RFL_4_FR and EFL_4_FE nanomembranes are oriented toward the internal ionic liquid region, enabling the formation of two chemically and electrostatically distinct interfacial environments. Visual inspection suggests the preferential accumulation of counterions near the charged peptide surfaces, indicating the onset of interfacial ionic organization driven by the intrinsic charge distribution of the nanomembranes. At this stage, however, Figure 3 is used primarily to confirm the structural stability, spatial orientation, and interfacial definition of the confined peptide/electrolyte model. Taken together, Figure 3 confirms that the two peptide nanomembranes preserve a stable and well-defined confined geometry throughout the production simulation. This structural persistence enables the quantitative analysis of ion-specific interfacial organization presented in the next section.

### Spontaneous Ionic-Liquid Layering at Intrinsically Charged Peptide Interfaces

After establishing the structural stability and general interfacial geometry of the confined peptide/electrolyte model, we next analyze the ionic-liquid organization in a quantitative manner. Figure 4 presents the mass density profiles along the *z* direction, enabling quantitative evaluation of ion accumulation, co-ion depletion, interfacial layering, and the spatial extension of the double-layer-like structures formed near the EFL_4_FE and RFL_4_FR surfaces.

**Figure 4:**
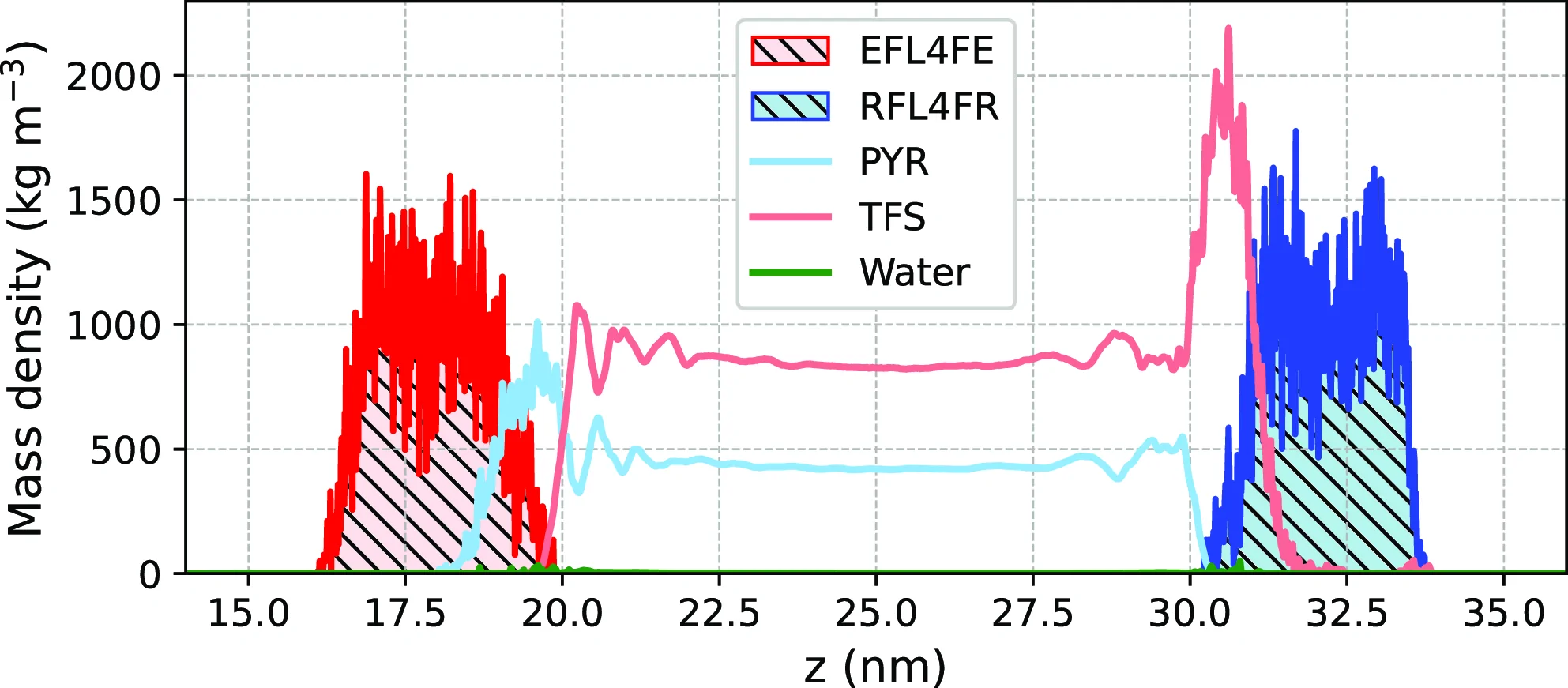
Mass density profiles along the *z* direction for EFL_4_FE, RFL_4_FR, [PYR]^+^, [TFSI]^−^, and water in the confined interfacial model. Counterion accumulation and ionic layering occur near both charged peptide surfaces, whereas the central electrolyte region exhibits a more homogeneous, bulk-like density.

The mass density profiles demonstrate preferential accumulation of [PYR]^+^ cations near the negatively charged EFL_4_FE surface and preferential accumulation of [TFSI]^−^ anions near the positively charged RFL_4_FR surface. At the same time, co-ion depletion and localized ionic layering are observed at both peptide/electrolyte boundaries, confirming the spontaneous formation of EDL-like structures. The different spatial organizations of [PYR]^+^, located approximately between 16 and 20 nm, and [TFSI]^−^, located approximately between 30 and 34 nm, further indicate that the interfacial response is not governed solely by electrostatic attraction, but also by ion size, molecular geometry, conformational flexibility, and charge delocalization. Thus, the confined ionic liquid acts as a chemically selective medium that translates the molecular charge pattern of the peptide membranes into asymmetric interfacial ionic architectures.

The structural differences between the positive and negative peptide/electrolyte interfaces are also reflected in the distinct spatial distributions of [PYR]^+^ and [TFSI]^−^ ions. The double-layer-like structure formed at the EFL_4_FE surface exhibits a larger effective thickness compared to the more compact ionic organization observed at the RFL_4_FR interface. This difference can be associated with the molecular characteristics of the electrolyte ions. The [TFSI]^−^ anion possesses greater charge delocalization and high conformational flexibility, allowing better accommodation near the positively charged RFL_4_FR surface. In contrast, [PYR]^+^ cations present a more rigid and bulkier structure, which partially reduces their packing capability near the negatively charged EFL_4_FE interface. As a result, the [TFSI]^−^ density profile exhibits sharper and more compact interfacial peaks, whereas the [PYR]^+^ distribution is comparatively more diffuse.

The respective contributions of the peptide surfaces and the ionic liquid can, therefore, be distinguished qualitatively. The intrinsic charges localized on the EFL_4_FE and RFL_4_FR nanomembranes provide an electrostatic driving force for counterion enrichment, co-ion depletion, and the development of EDL-like interfacial regions. Without these oppositely charged peptide surfaces, the charge-selective accumulation observed here would not be expected to arise from the same electrostatic mechanism, although chemically specific adsorption could still occur at an uncharged interface. The physicochemical properties of [PYR]­[TFSI] determine how the peptide-induced electrostatic response is spatially expressed. The [PYR] cation exhibits a relatively broad interfacial distribution near the negatively charged EFL_4_FE surface, whereas [TFSI] forms a narrower and more pronounced accumulation region near the positively charged RFL_4_FR interface. This difference may be associated with the molecular size, geometry, conformational flexibility, and charge distribution of the two ions, although explicit orientational and conformational analyses are required to establish their individual contributions. The oscillatory interfacial profiles are consistent with the correlated ionic layering commonly observed in neat ionic liquids, in contrast to the diffuse screening characteristic of dilute electrolytes. Thus, the peptide surface charges spatially direct the EDL-like organization, whereas the molecular properties and collective behavior of [PYR]­[TFSI] contribute to its observed thickness, compactness, asymmetry, and decay. Because only one ionic liquid was considered, these contributions cannot be quantitatively separated, and comparative simulations with alternative electrolytes would be required to determine their relative importance.

Another important aspect observed in Figure 4 concerns the central region of the confined ionic liquid, located approximately between 21 and 29 nm. In this region, the density profiles become nearly homogeneous, indicating a more bulk-like behavior of the ionic liquid. The absence of strong oscillations in the center of the confined domain demonstrates that the double-layer-like structures remain primarily localized near the peptide interfaces, although part of the structural organization induced by the charged surfaces still propagates into the electrolyte interior. This result is particularly relevant because it indicates that the membrane separation distance, approximately 12 nm, is sufficient to avoid significant overlap between the interfacial ionic layers, enabling an independent characterization of the positive and negative peptide/electrolyte interfaces.

The presence of small amounts of water within the system also influences the interfacial organization of the confined electrolyte. Although the water density remains relatively low throughout the simulation box, a slight enrichment is observed near the peptide interfaces. This behavior suggests that water molecules may partially mediate interactions between polar peptide groups and electrolyte ions, contributing to the stabilization of the interfacial ionic structure. In addition, residual water molecules may facilitate local structural rearrangements in the interfacial region, especially through hydrogen-bonding interactions involving peptide terminal groups and ionic-liquid species. Hydrogen-bond analyses obtained from simulations of the peptide nanomembranes in [PYR]­[TFSI] are presented in Figure S3 of the Supporting Information and support the persistence of the peptide–peptide hydrogen-bond network in the ionic-liquid environment.

Overall, the mass density profiles show that the EFL_4_FE/[PYR]­[TFSI]/RFL_4_FR model forms well-defined peptide/electrolyte interfaces, with ionic distributions consistent with organization driven by the intrinsic peptide surface charges and modulated by ion-specific properties and collective ionic behavior. The distinct interfacial distributions of [PYR]^+^ and [TFSI]^−^ indicate that differences in molecular size, geometry, conformational flexibility, and charge distribution may contribute to the local structure of the EDL-like regions. These results provide a molecular-level basis for interpreting the electrostatic potential profiles discussed in the next section, where the consequences of this ionic organization for interfacial charge separation and the spatial variation of the electrostatic potential are examined.

### Electrostatic Potential Profiles and Double-Layer-like Organization

Figure 5 presents the electrostatic potential profile along the *z*-direction for [PYR]­[TFSI] confined between the EFL_4_FE and RFL_4_FR nanomembranes. The potential remains approximately constant in the central, bulk-like electrolyte region and varies sharply near the two peptide/electrolyte interfaces. This spatial behavior connects the ion-density organization described in the previous session to the trajectory-averaged electrostatic response generated by the intrinsically charged peptide surfaces.

**Figure 5:**
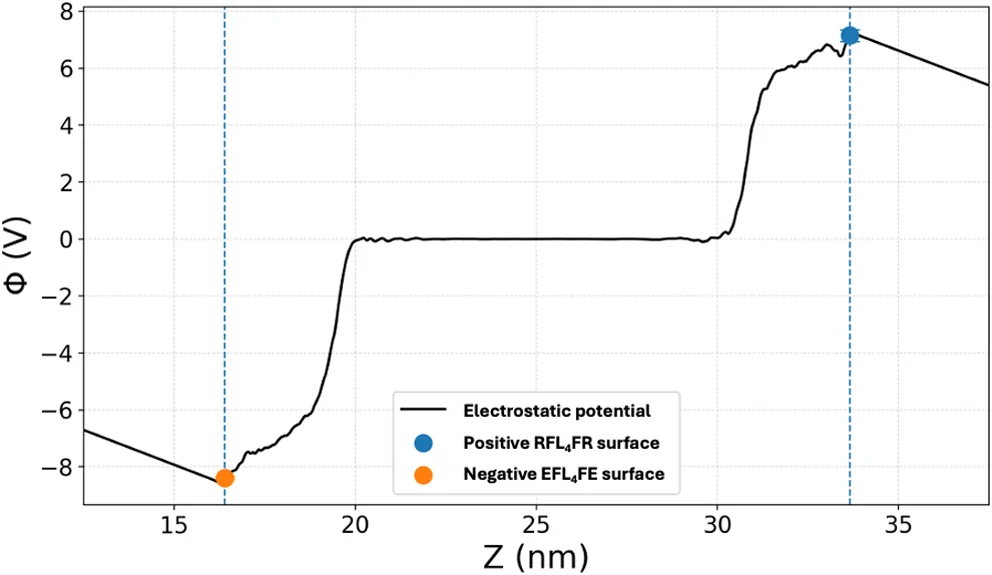
Electrostatic potential Φ, in V, profile along the *Z* direction, in nm, for the confined EFL_4_FE/[PYR]­[TFSI]/RFL_4_FR model, obtained by numerical integration of the one-dimensional Poisson equation. The potentials at *Z* ≈ 16.40 and 33.66 nm are Φ^–^ = −8.39 ± 0.06 V and Φ^+^ = +7.15 ± 0.21 V, respectively, yielding ΔΦ = 15.54 ± 0.22 V. The reported ΔΦ is a model-dependent electrostatic descriptor obtained within the classical nonpolarizable fixed-charge framework and should not be interpreted as an experimentally accessible operating voltage. Block-resolved electrostatic potential profiles used to assess the statistical stability of these values are provided in Figure S4 of the Supporting Information.

The procedure used to calculate the electrostatic potential follows the same methodology commonly adopted in previous molecular dynamics studies of electrochemical double-layer systems, in which the electrostatic potential is obtained through numerical integration of the one-dimensional Poisson equation along the direction normal to the charged interfaces. In previous studies involving biodegradable peptide nanosheets, the electrostatic potential across the system was also calculated from the spatial charge distribution, and the half-cell potentials were defined as the difference between the charged interface and the bulk electrolyte potential.^7^ The same computational approach is applied here, with the center of the confined electrolyte chosen as the zero-potential reference because this region exhibits an approximately homogeneous, bulk-like charge distribution. The absolute value of an electrostatic potential obtained from Poisson integration is defined only up to an arbitrary additive constant; therefore, assigning zero potential to a different spatial region would shift the entire profile vertically and would change the reported individual values of Φ^–^ = −8.39 ± 0.06 and Φ^+^ = +7.15 ± 0.21 V. By contrast, the potential difference between two fixed positions in the same calculated profile is invariant with respect to this additive shift. Nevertheless, the reported ΔΦ = 15.54 ± 0.22 V remains dependent on the selected spatial evaluation points, here the two external membrane regions, as well as on the planar averaging, integration procedure, charge model, and boundary conditions adopted in the simulation. The relatively small block-to-block variations indicate that the principal features of the electrostatic potential profile are statistically stable over the analyzed production trajectory. Consistent behavior is also observed for the block-resolved profiles (Figure S4), supporting the temporal convergence of the interfacial observables used in the present analysis.

This point is particularly important because the ionic liquid is present only within the internal region between the charged faces of the peptide nanomembranes. Therefore, the electrostatic potential should not be interpreted solely at the surface directly in contact with the electrolyte but also across the membrane structure, including the opposite external face. In the present framework, this external face should be viewed as a structurally accessible region through which the electrostatic response generated at the internal peptide/electrolyte interface can be evaluated. In a future device-oriented architecture, this region could be explored as a possible coupling site for current collectors, although explicit modeling of electronic conduction and electrode-collector coupling would be required to validate such a design. Consequently, the potential observed at the external sides of the membranes represents the integrated electrostatic response of the peptide nanomembranes: the internal charged surface organizes the electrolyte, while the resulting electrostatic perturbation remains reflected in the potential calculated across the peptide region.

On the left side of Figure 5, associated with the EFL_4_FE membrane, the electrostatic potential reaches approximately Φ^–^ = −8.39 ± 0.06 V at *Z* ≈ 16.40 nm. This value corresponds to the effective potential of the region associated with the negatively charged peptide interface relative to the electrolyte bulk. On the right side, associated with the RFL_4_FR membrane, the electrostatic potential reaches approximately Φ^+^ = +7.15 ± 0.21 V at *Z* ≈ 33.66 nm. The total electrostatic potential difference between the two external membrane regions is therefore ΔΦ = 15.54 ± 0.22 V. Within the present classical nonpolarizable fixed-charge model, this unusually large value reflects the strong electrostatic response generated by the high intrinsic surface charge density of the peptide membranes and the ionic organization of the confined electrolyte. The intrinsic surface charge density, estimated in Table 1 as approximately ±3.24 e/nm^2^, is substantially larger than those typically employed in conventional models of planar carbon or graphene-based EDL systems. In physical terms, the reported values describe the trajectory-averaged electrostatic potential variation obtained by integrating the planar charge density distribution of the simulated peptide/electrolyte system. The individual potentials assigned to the two membrane regions depend on the choice of the zero-potential reference, whereas the difference between the same two spatial positions is invariant under an arbitrary additive shift of the complete potential profile.

The comparison with previous peptide-based systems is particularly useful for contextualizing the electrostatic regime accessed in the present model. In the work by Colherinhas, Malaspina, and Fileti on biodegradable peptide-based electrochemical interfaces, the EFL_4_FE and RFL_4_FR membranes were constructed with an approximate area of 17.8 nm^2^, resulting in a surface charge density of 57.6 μC/cm^2^, described by the authors as high and approximately 1 order of magnitude larger than values typically employed in electrochemical supercapacitor simulations.^7^ Converting this value, 57.6 μC/cm^2^ corresponds to approximately 3.6 e/nm^2^, which is close to the surface charge density employed in the present model. This comparison indicates that the present confined peptide/electrolyte model belongs to a high surface-charge-density regime characteristic of intrinsically charged peptide nanomembranes, rather than to the lower charge density regime commonly adopted for externally polarized planar carbon-based electrodes. The physical significance of this high value must be understood in the context of the molecular architecture of the peptide assemblies. Each nanolayer contains a dense, periodic arrangement of charged terminal residues, with formal charges concentrated within a relatively small interfacial area. Consequently, values in the range of 3.2–3.6 e/nm^2^ are internally consistent with the modeled peptide stoichiometry and the compact packing of the nanolayers; they are also comparable to the value obtained in the previous atomistic model of the same EFL_4_FE/RFL_4_FR assemblies.^7^ The underlying nanolayer geometry is based on previously characterized self-assembled structures, the dimensions of which were found to be compatible with experimental SAXS measurements.^14^ However, the surface charge density presented here should not be interpreted as a direct experimental measurement of the effective charge exposed under all solution conditions. In an experimental system, factors such as pH, specific ion binding, counterion condensation, structural defects, membrane hydration, and variations in molecular packing could reduce or redistribute the effective interfacial charge. Thus, the reported value represents the fully charged, structurally ordered state defined by the current atomistic model.

In graphene-based systems containing ionic liquids, the surface charge densities employed in simulations are generally much smaller. For instance, in graphene-based supercapacitor models containing amino acid ionic liquids, maximum potential values ranging from approximately 2.095 to 2.353 V were reported for systems subjected to surface charge densities of ±4.81 μC/cm^2^.^39^ This density corresponds to approximately ±0.30 e/nm^2^, nearly 1 order of magnitude smaller than the charge density employed in the present peptide-based system. Here, the peptide nanomembranes exhibit intrinsic surface charge densities approximately ten times larger than those used in graphene-based simulations. Accordingly, the surface charge density of approximately ±3.24 e/nm^2^ generates a much larger electrostatic potential difference, with ΔΦ = 15.54 ± 0.22 V, as observed in Figure 5. This comparison does not imply a direct linear scaling between charge density and electrostatic potential, since confined ionic liquids are strongly affected by interfacial saturation, ion packing, overscreening, and ionic correlations. Rather, it supports the interpretation that highly localized peptide surface charges promote strong ionic-liquid polarization and interfacial electrostatic separation within the simulated fixed-charge framework. In addition, because the electrostatic potential is obtained from a trajectory-averaged planar charge density profile and is defined relative to a chosen reference region, the numerical magnitude of the calculated potential variation is specific to the present model and analysis protocol and should not be transferred directly to experimental voltage scales.

Although the high intrinsic surface charge density is the principal origin of the large electrostatic potential difference obtained in the present fixed-charge model, the magnitude and spatial form of the electrostatic response are also influenced by peptide-specific interfacial features. Unlike an atomically homogeneous planar electrode, the peptide surfaces contain chemically localized COO^–^ and positively charged terminal groups embedded within a heterogeneous supramolecular environment. Because these charges are assigned to specific molecular sites and cannot redistribute electronically within the nonpolarizable classical force-field description, they generate spatially localized electric fields that are screened primarily through ionic rearrangement in the confined electrolyte. In addition, nanoscale surface heterogeneity, local roughness, peptide conformational fluctuations, and the distribution of polar and aromatic groups determine the closest approach, orientation, and packing of [PYR]^+^ and [TFSI]^−^ ions. Hydrogen-bonding interactions involving peptide terminal groups, residual water, and ionic-liquid species may further stabilize particular interfacial configurations, while the β-sheet hydrogen-bond network, hydrophobic leucine packing, and aromatic interactions preserve the supramolecular arrangement that maintains the charge pattern throughout the simulation.

Therefore, the intrinsic surface charge density is expected to set the overall scale of the electrostatic potential, whereas charge localization, molecular heterogeneity, hydrogen bonding, supramolecular organization, and ion-specific packing are expected to influence interfacial screening, potential asymmetry, and detailed spatial profile. Because no chemically homogeneous reference surface with the same charge density was simulated, the individual contributions of these surface-specific molecular factors could not be quantitatively isolated in the present study.

The sensitivity of the interfacial response to surface charge density was also not explicitly quantified because only one predefined protonation state, corresponding to the fully charged peptide model considered here, was simulated. On physical grounds, reducing the fraction of ionized terminal groups would generally be expected to decrease counterion enrichment, weaken co-ion depletion, reduce the amplitude of charge density oscillations, and lower the electrostatic potential gradient. However, these responses are not expected to vary linearly with surface charge density because ionic liquid screening is influenced by molecular packing, interfacial saturation, overscreening, and strong electrostatic correlations. The qualitative conclusion that oppositely charged peptide surfaces direct ion-specific interfacial organization may remain valid over a finite range of surface charge densities, whereas the quantitative magnitudes of ionic layering, potential differences, and charge-to-potential descriptors are necessarily dependent on the peptide’s charge state. Establishing this dependence will require additional simulations in which the predefined peptide protonation states, and consequently the effective surface charge densities, are systematically varied.

Consistent with this interpretation, the asymmetry between the two sides of the electrostatic potential profile reflects the combined effects of peptide-specific surface chemistry and the distinct ways in which [PYR]^+^ and [TFSI]^−^ ions pack and interact at the intrinsically charged interfaces. The negative potential associated with the EFL_4_FE interface exhibits a slightly larger magnitude than the positive potential associated with the RFL_4_FR interface. Previous studies involving peptide nanosheets and the [Ch]­[Gly] ionic liquid demonstrated that interfacial asymmetry can emerge from the combined effects of peptide surface chemistry and structural differences between the ionic-liquid cation and anion.^7^ Similarly, in the present system, [PYR]^+^ cations and [TFSI]^−^ anions possess different sizes, geometries, conformational flexibilities, and charge distributions, resulting in ionic layers with different thicknesses and compactness at each peptide/electrolyte interface.

An important difference in the electrostatic potential scale is observed when comparing the present system with the previous peptide-based biodegradable supercapacitor model.^7^ In the biodegradable EFL_4_FE/[Ch]­[Gly]/RFL_4_FR system, the authors obtained Φ^+^ = 1.8 V, Φ^–^ = −1.3 V, and ΔΦ = 3.1 V, despite the high surface charge density stabilized at the peptide interfaces.^7^ In the present model, however, the total electrostatic potential difference reaches ΔΦ = 15.54 ± 0.22 V. These values indicate that replacing [Ch]­[Gly] with [PYR]­[TFSI] substantially alters ionic screening and the trajectory-averaged charge density distribution. Nevertheless, even this comparison must remain restricted to the relative electrostatic response obtained within the respective classical simulation models. Differences in electrolyte composition, simulation geometry, water content, ionic packing, potential reference definition, and spatial evaluation points prevent the numerical values from being interpreted as directly comparable experimental cell voltages.

The relationship between the calculated electrostatic potential profile and experimentally measurable electrochemical voltages requires a clear distinction. Experimental operating voltages and electrochemical stability windows are defined under specific electrode, reference, and thermodynamic conditions and include electronic and chemical contributions that are not represented in the present molecular model.^1^ Therefore, the total electrostatic potential difference obtained in the present simulations should not be interpreted as an experimentally accessible operating voltage, cell voltage, or electrochemical stability window. The value of ΔΦ = 15.54 ± 0.22 V is derived from the one-dimensional Poisson integration of blocks of the trajectory-averaged charge density profiles within a classical, nonreactive, nonpolarizable fixed-charge framework. This representation does not include electronic charge redistribution, constant-potential electrode behavior, explicit electronic polarization, proton transfer, electrolyte decomposition, electron transfer, bond-breaking or bond-forming events, or faradaic reactions, all of which could substantially modify the electrochemical response of a real system. Consequently, the calculated ΔΦ should be interpreted exclusively as a model-dependent descriptor of the strength and spatial extent of interfacial electrostatic polarization sustained by the peptide charge distribution and the confined ionic liquid, rather than as a quantitatively transferable operational voltage.

From the electrostatic perspective, the potential analysis allows an estimation of the effective interfacial response through the relation *C* = σ/ΔΦ, using the trajectory-block averages. For σ = ±3.24 e/nm^2^, corresponding to approximately 0.519 C/m^2^, and the half-cell potentials extracted from Figure 5, effective capacitance-like values of approximately *C*_–_ = 6.19 ± 0.04 μF/cm^2^ and *C*_+_ = 7.26 ± 0.21 μF/cm^2^ were obtained for the negative and positive interfaces, respectively, resulting in an overall series-equivalent value of approximately 3.34 ± 0.05 μF/cm^2^. These values should be interpreted as capacitance-like descriptors of interfacial electrostatic response, rather than as experimentally accessible operational capacitances. In the present peptide-based system, the interfacial response arises from intrinsically charged nanomembranes whose molecular structure, protonation state, and residual surface charges promote ionic-liquid polarization and EDL-like organization under self-organized conditions. Nevertheless, the estimated values remain within the same order of magnitude commonly reported for graphene/ionic-liquid supercapacitor models, where total interfacial capacitances frequently range between 3 and 8 μF/cm^2^. Similar values have also been reported for graphene/[EMIM]­[BF_4_] systems, in which capacitances of approximately 3–4 μF/cm^2^ were obtained under substantially lower surface charge densities and smaller electrostatic potential differences.^7^

A direct comparison with our previous peptide-based biodegradable supercapacitor model reported in ACS Omega^7^ further highlights the distinct electrostatic regime established in the present system and, more importantly, provides a refined physical interpretation of peptide-based electrostatic interfaces. In ref 7, peptide nanosheets combined with amino acid ionic liquids exhibited total capacitances close to 18.7 μF/cm^2^, electrostatic potential differences of approximately 3.1 V, and stored electrostatic energies on the order of 90 J/cm^3^, values comparable to conventional graphene-based ionic-liquid supercapacitor models. Importantly, the peptide systems investigated in the previous study were constructed following essentially the same physical principles employed here, including intrinsically charged peptide nanosheets and spontaneous ionic-liquid organization at the interfaces. In the present system, the values of *C* and ΔΦ may be combined through the classical capacitor expression only as a formal internal descriptor of the fixed-charge electrostatic state. Using the trajectory-block averages and an effective interfacial thickness of 10 nm, corresponding to the distance between the membrane surfaces, the resulting energy-like density is approximately 404 ± 6 J/cm^3^. This value should not be interpreted as stored, extractable, or experimentally measurable energy density. The present simulations do not describe an externally driven charging process, electrical work supplied by a source, reversible discharge, or coupling to a load. Therefore, this energy-like quantity reflects only the mathematical combination of the calculated fixed-charge potential profile and the derived capacitance-like descriptor. It may be used for comparison with the previous peptide model only if both quantities are explicitly understood as model-dependent descriptors obtained under related computational assumptions.^7^

Here, the amplified electrostatic state emerges from the intrinsic molecular charge distribution stabilized within the peptide nanomembranes and from the highly organized double-layer-like structures formed in the confined [PYR]­[TFSI] electrolyte. Therefore, the present work not only extends the electrostatic intensity previously observed in peptide-based systems^7^ but also supports a conceptual framework in which high interfacial polarization can emerge directly from supramolecular organization and confined ionic-liquid structuring.

This interpretation also highlights the importance of the molecular nature of the charged interfaces. In planar graphene or gold systems, EDL-like formation occurs on rigid and relatively well-defined surfaces with limited ionic semipermeability. In contrast, peptide-based surfaces may be rougher, more flexible, and chemically heterogeneous, allowing for differentiated ionic access to localized charged sites. Previous studies have shown that ionic semipermeation within peptide surfaces can enhance access to charged sites and increase the interfacial capacitance, representing one of the main reasons why peptide-based systems exhibited larger capacitances than analogous systems employing planar metallic electrodes.^7^

The possible connection of the peptide nanomembranes to current collectors or their use as electrostatic transduction elements should be regarded as a hypothesis for future device design. Figure 5 shows that the trajectory-averaged electrostatic potential varies across the peptide regions and presents distinct values at the selected external-surface positions of EFL_4_FE and RFL_4_FR. Within the classical fixed-charge model, this result indicates that the electrostatic perturbations associated with the peptide/electrolyte interfaces are reflected throughout the corresponding peptide regions rather than being restricted to the immediate electrolyte-facing interfaces. However, this spatially extended equilibrium profile should not be interpreted as evidence of electronic conduction, signal transduction, or coupling to an external circuit. Evaluation of these functions would require models incorporating appropriate conductive supports or current collectors, externally applied electrical conditions, and explicit descriptions of the relevant electronic or ionic transport processes. Therefore, the present results support only the conclusion that the interfacial electrostatic perturbation remains spatially expressed across the simulated peptide structures. Whether this behavior can be exploited in electrostatic transduction or operational device architectures remains a subject of future investigation.

### From Intrinsic Peptide Charge to Biomolecular Electrostatic Function

One of the central implications of the present results is that the peptide nanomembranes should be interpreted as intrinsically charged biomolecular electrostatic interfaces rather than as conventional externally polarized electrodes. In classical EDL systems based on graphene, porous carbon, or metallic surfaces, interfacial polarization is usually generated by assigning fixed charges to electrode atoms or by imposing constant-potential conditions. In contrast, the electrostatic response observed here originates directly from the molecular composition, protonation state, and supramolecular organization of the EFL_4_FE and RFL_4_FR nanosheets. The EFL_4_FE membrane exposes negatively charged carboxylate groups, whereas the RFL_4_FR membrane presents positively charged guanidinium-containing groups. Therefore, the physical sequence governing the present system is molecular charge localization → ionic-liquid organization → interfacial electrostatic polarization, rather than the conventional externally imposed electrode charge → electrolyte polarization mechanism.

This distinction places the present system closer to charged biomolecular and biological interfaces than to ideal carbon-based EDLC electrodes. In biological membranes, proteins, peptides, and colloidal soft interfaces, surface charges naturally emerge from the molecular composition and organize surrounding ions without the need for externally imposed polarization. Similarly, in the present nanosheets, the peptide architecture defines the local electrostatic environment and spontaneously drives counterion accumulation, ionic layering, and an EDL-like organization in the confined [PYR]­[TFSI] electrolyte. The resulting interface is therefore an emergent property of supramolecular self-assembly, localized charge distribution, and ionic liquid confinement. In this sense, the peptide membranes behave not only as passive supports for electrolyte adsorption but also as intrinsically charged molecular domains that shape the equilibrium electrostatic landscape of the confined ionic medium.

The interfacial organization observed here is unlikely to be exclusive to the EFL_4_FE and RFL_4_FR sequences, although these peptides provide a structurally well-defined realization of this behavior. More generally, similar self-organized electrostatic interfaces may emerge in other peptide or biomolecular assemblies that satisfy three principal conditions: the presence of stable and solvent-accessible molecular charges, the ability to self-assemble into persistent interfacial architectures, and the spatial localization of these charges at surfaces accessible to the electrolyte. Peptide amphiphiles, charged fibrils, nanosheets, and other biomolecular supramolecular interfaces may therefore exhibit related counterion enrichment, co-ion depletion, and EDL-like organization when their charged groups remain exposed and structurally organized. Nevertheless, the magnitude, asymmetry, and spatial extent of the response are expected to remain dependent on the peptide sequence, supramolecular structure, electrolyte composition, and confinement conditions.

In the present EFL_4_FE/RFL_4_FR system, the bolaamphiphilic architecture spatially separates the charged terminal groups from the hydrophobic L_4_ core. Phenylalanine-mediated interactions, β-sheet hydrogen bonding, and leucine packing contribute to the stabilization of extended EFL_4_FE and RFL_4_FR nanosheets whose electrolyte-facing surfaces carry opposite signs of charge. These structural features produce substantial effective surface charge densities and maintain interfacial asymmetry over the simulated time scale. Other peptide assemblies may reproduce the same general behavior, but their effective charge density, surface morphology, predefined protonation state, structural stability, ion accessibility, and electrolyte environment will determine the strength, asymmetry, and spatial extent of the resulting electrostatic interface. Comparative simulations involving alternative peptide sequences, supramolecular morphologies, protonation states, and electrolyte compositions will therefore be required to establish the quantitative transferability of the present findings.

From a broader perspective, these findings provide a basis for future investigations of biodegradable electrostatic materials, soft iontronic interfaces, and molecularly programmable energy-related systems in which controlled ionic organization and electrostatic potential gradients may be functionally relevant. Translating the mechanism observed here into an operational device will nevertheless require additional computational and experimental studies addressing systematic variation of peptide charge states, electrolyte composition, external polarization, conductive coupling, relevant electronic or ionic transport processes, and device architecture.

## Conclusion

In conclusion, fully atomistic molecular dynamics simulations were used to characterize the structural, interfacial, and electrostatic behaviors of the ionic liquid [PYR]­[TFSI] confined between intrinsically charged EFL_4_FE and RFL_4_FR peptide nanomembranes. The simulations directly demonstrate that the peptide nanosheets preserve their supramolecular integrity and maintain spatially organized oppositely charged interfaces throughout the unrestricted production trajectory. These interfaces induce selective counterion accumulation, co-ion depletion, asymmetric ionic layering, charge density oscillations, and EDL-like organization within the confined ionic liquid.

The observed interfacial organization results from the combined influence of the intrinsic peptide surface charge and the ion-specific properties of [PYR]­[TFSI]. The localized charges of the EFL_4_FE and RFL_4_FR nanomembranes provide the primary electrostatic driving force for ionic segregation, whereas the size, molecular geometry, conformational flexibility, charge delocalization, and correlation behavior of the ionic liquid species determine the compactness, asymmetry, and spatial extension of the interfacial layers. The high intrinsic surface charge density establishes the overall scale of the calculated electrostatic response, while peptide surface heterogeneity, localized molecular charge, hydrogen bonding, supramolecular stability, and ion-specific packing modulate the detailed forms of the density and electrostatic potential profiles.

These findings are consistent with the broader literature on peptide-based supramolecular materials, which highlights their structural adaptability, self-assembly capability, and molecularly tunable interfacial properties.^13^ They are also conceptually related to investigations of charged soft and hydrogel-based interfaces, in which spatially organized molecular charges promote ion accumulation and EDL formation,^32^ and to studies of EDL-controlled interfaces in which ionic organization governs strong interfacial electrostatic coupling.^40^ These comparisons provide a broader physical context for the present results but should not be interpreted as evidence that the peptide nanomembranes already perform iontronic, electronic, or electrostatic transduction functions.

The strong ionic layering and oscillatory interfacial organization observed here are further compatible with previous studies showing that ionic liquids confined near charged interfaces exhibit nonclassical screening, overscreening, charge density oscillations, and strong ion–ion correlations. In particular, the pronounced electrostatic potential gradients obtained in the present model are consistent with the general conclusion that strong surface-charge localization and confinement can intensify electrostatic ordering and modify the screening response of ionic-liquid interfaces.^41,42^ Nevertheless, the calculated potential difference of approximately ΔΦ = 15.54 ± 0.22 V must be interpreted exclusively as a model-dependent descriptor of interfacial electrostatic polarization within the classical, nonreactive, nonpolarizable fixed-charge framework.

The adopted model does not include electronic charge redistribution, constant-potential electrode behavior, explicit electronic polarization, proton transfer, electrolyte decomposition, electron transfer, Faradaic reactions, current collectors, or coupling to an external circuit. Consequently, the calculated electrostatic potential difference and capacitance-like quantities should not be interpreted as experimentally accessible operating voltages, device-level capacitances, electrochemical stability windows, or direct measures of practical energy storage performance. Likewise, the spatial extension of the trajectory-averaged electrostatic potential across the peptide structures demonstrates the distribution of the calculated electrostatic response within the model but does not by itself establish electronic conduction, current collection, or signal transduction.

Overall, the present study provides molecular-level evidence that intrinsically charged peptide nanomembranes can direct the organization of dense ionic liquids under confinement. The principal contribution of this work is therefore the characterization of the relationship among localized peptide surface charge, supramolecular stability, membrane flexibility, ion-specific layering, and interfacial electrostatic polarization. Although the general mechanism may extend to other charged and structurally persistent peptide assemblies, its quantitative expression is expected to depend on the peptide sequence, protonation state, supramolecular morphology, surface charge density, electrolyte identity, and ion accessibility. In particular, the values reported here correspond to the fully ionized peptide state adopted in the simulations; different experimental protonation conditions or degrees of charge compensation would be expected to modify the quantitative electrostatic response. Possible applications in iontronics, electrostatic transduction, energy-related interfaces, and externally coupled devices remain future directions that will require additional computational and experimental studies explicitly addressing electronic transport, external polarization, charge transfer processes, current collector coupling, and operational device architectures.

## Supplementary Material



## Notes

Mass and charge density profiles of the isolated peptide/ionic-liquid interfaces; potential energy evolution; peptide–peptide hydrogen-bond analyses; and block-resolved electrostatic potential profiles (PDF)
