# Supplementary material for: Intrinsically Charged Peptide Nanomembranes as Self-Organized Electrostatic Interfaces in Confined Ionic Liquids
Source: Langmuir. 2026 Sep 16;42(43):31703–16. doi: 10.1021/acs.langmuir.6c03513 (PMC13738790; doi:10.1021/acs.langmuir.6c03513)
Supplement: Supplementary file 1 [file la6c03513_si_001.pdf]

SUPPORTING INFORMATION

Intrinsically Charged Peptide Nanomembranes as Self-Organized Electrostatic Interfaces in Confined Ionic Liquids

Karinna Mendanha[1,2], Tertius Lima Fonseca[2], and Guilherme Colherinhas[2]*

[1] Departamento de Física, CEPAE, Universidade Federal de Goiás, 74690-900, Goiânia, Goiás, Brazil.
[2] Instituto de Física, Universidade Federal de Goiás, 74690-900, Goiânia, Goiás, Brazil.
** Corresponding author. E-mail address: gcolherinhas@ufg.br*

Supporting Information Contents

Figure S1 (p. S2)
Mass and electric charge density profiles of the peptide membranes in ionic liquid.

Figure S2 (p. S3)
Convergence of the potential energy of the EFL_4FE and RFL_4FR structures in ionic liquid.

Figure S3 (p. S4)
Hydrogen-bond analysis for peptide–peptide interactions.

Figure S4 (p. S5)
Block-resolved electrostatic potential profiles for the confined EFL_4FE/[PYR][TFSI]/RFL_4FR system.

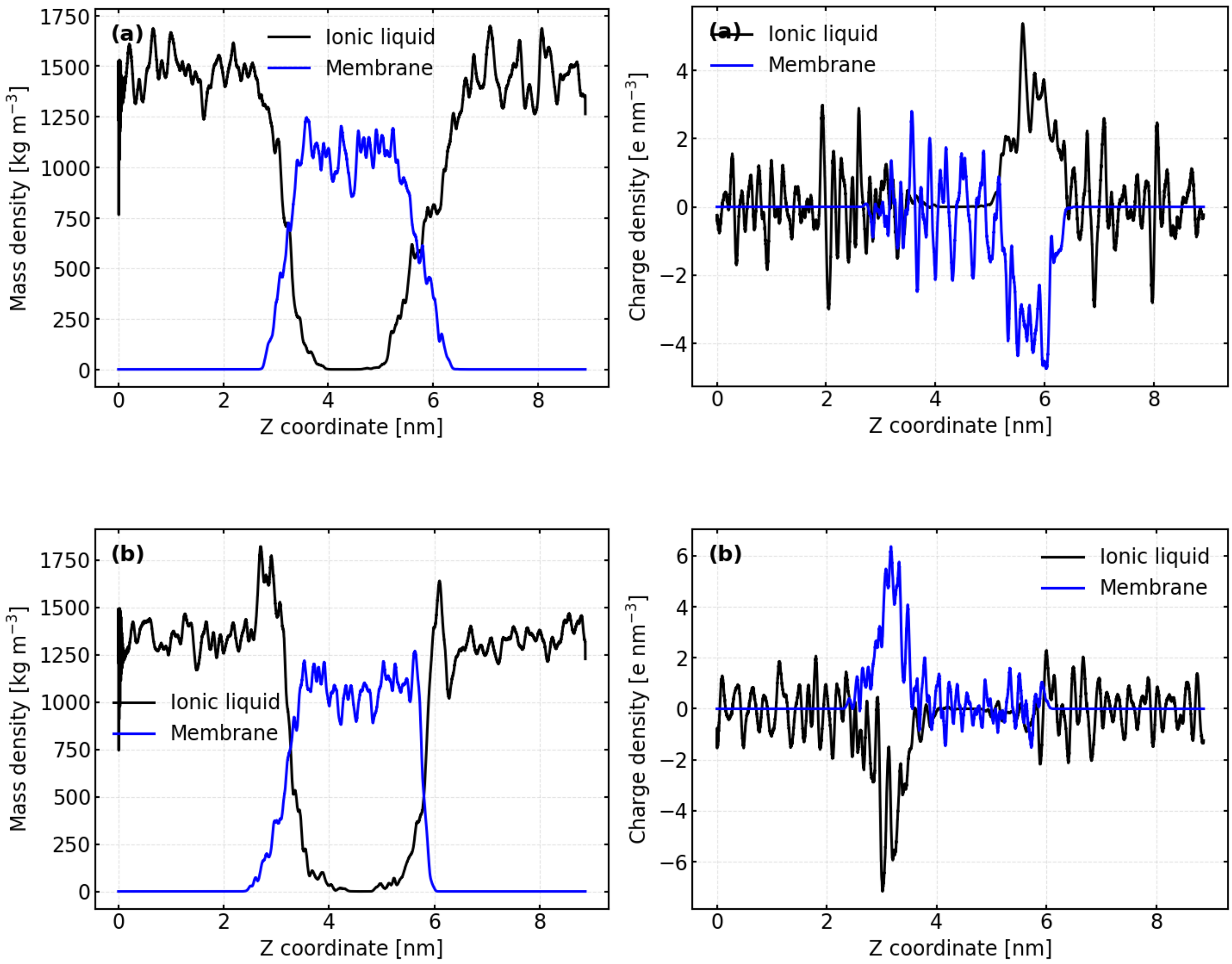

Figure S1: Representation of the mass [in kg/m³] and electric charge [in e/nm³] density profiles of peptide membranes in ionic liquid along the *Z* coordinate [in nm]. In (a), the EFL_4FE membrane is shown, where the blue curve corresponds to the membrane density and the black curve represents the ionic liquid density. In (b), the RFL_4FR membrane is presented, in which the blue curve corresponds to the membrane density and the black curve represents the ionic liquid density.

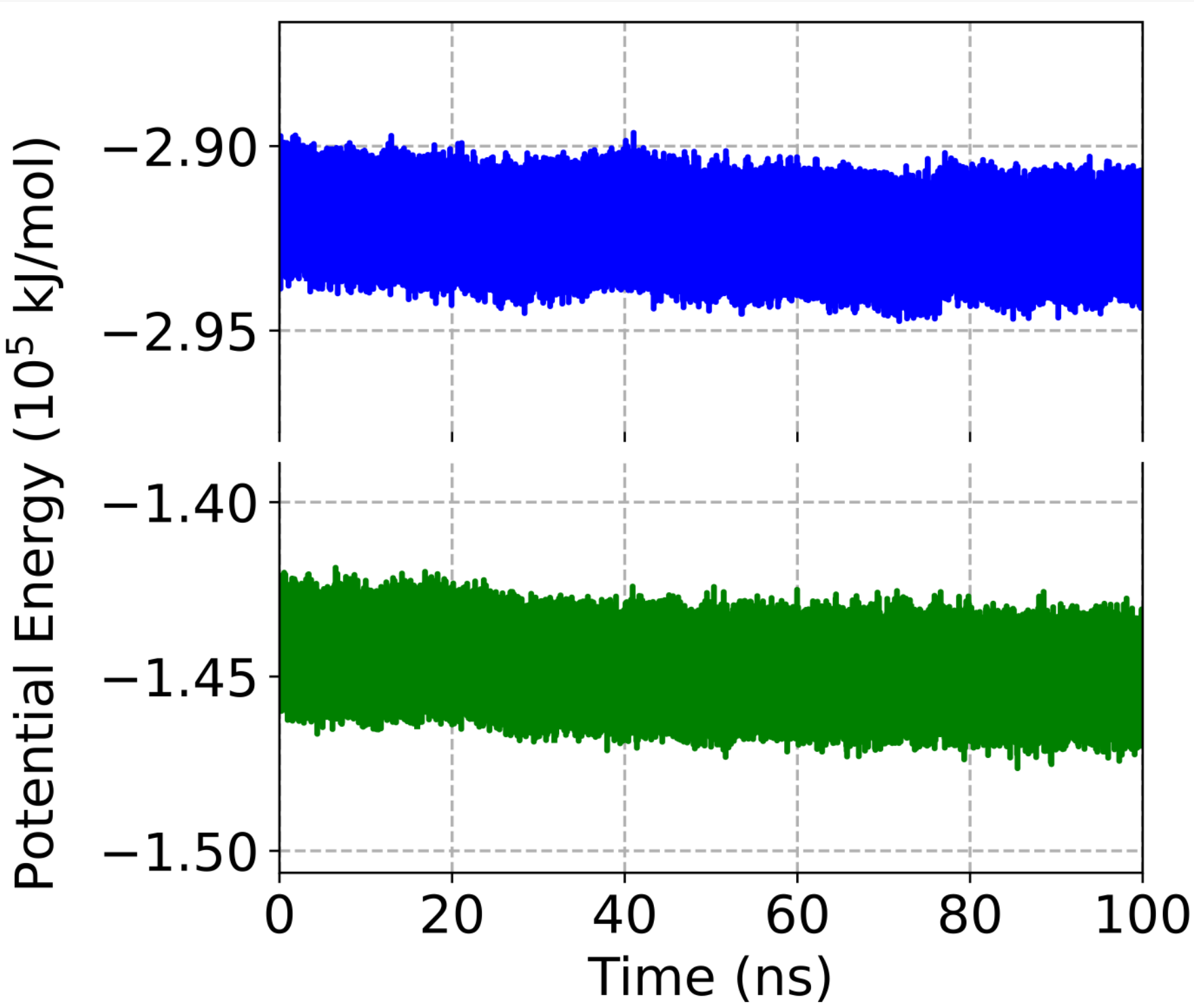

Figure S2: Demonstration of the convergence of the potential energy of the EFL_4FE and RFL_4FR structures in ionic liquid. Results obtained using NPT MD-simulations.

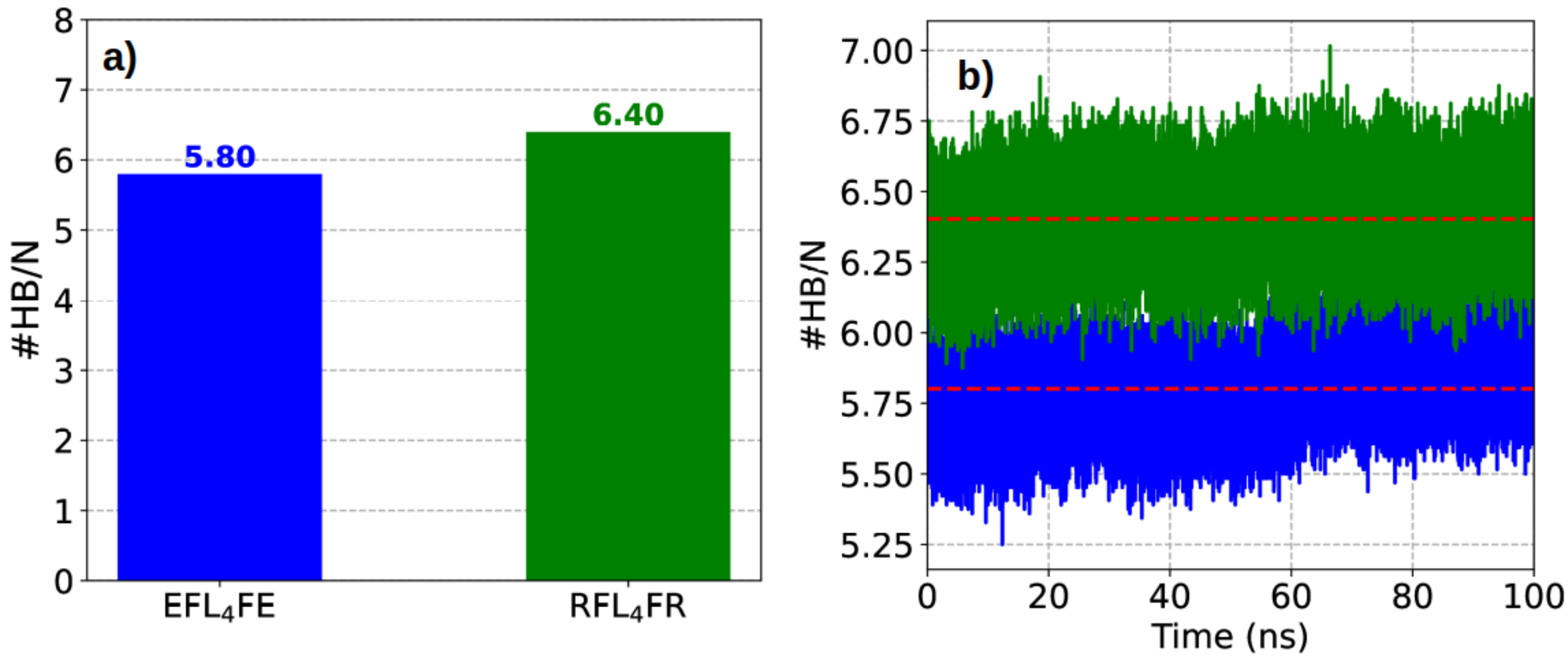

Figure S3: In Figure (a), the average number of hydrogen bonds per residue (HB/N) for peptide–peptide interactions in the simulations of peptide membranes in ionic liquid is presented. In (b), the temporal behavior of the hydrogen bonds associated with peptide–peptide interactions throughout the simulation is shown. The blue curve represents the EFL_4FE membrane, while the green curve corresponds to the RFL_4FR membrane. The red line indicates the average hydrogen bond value during the simulation.

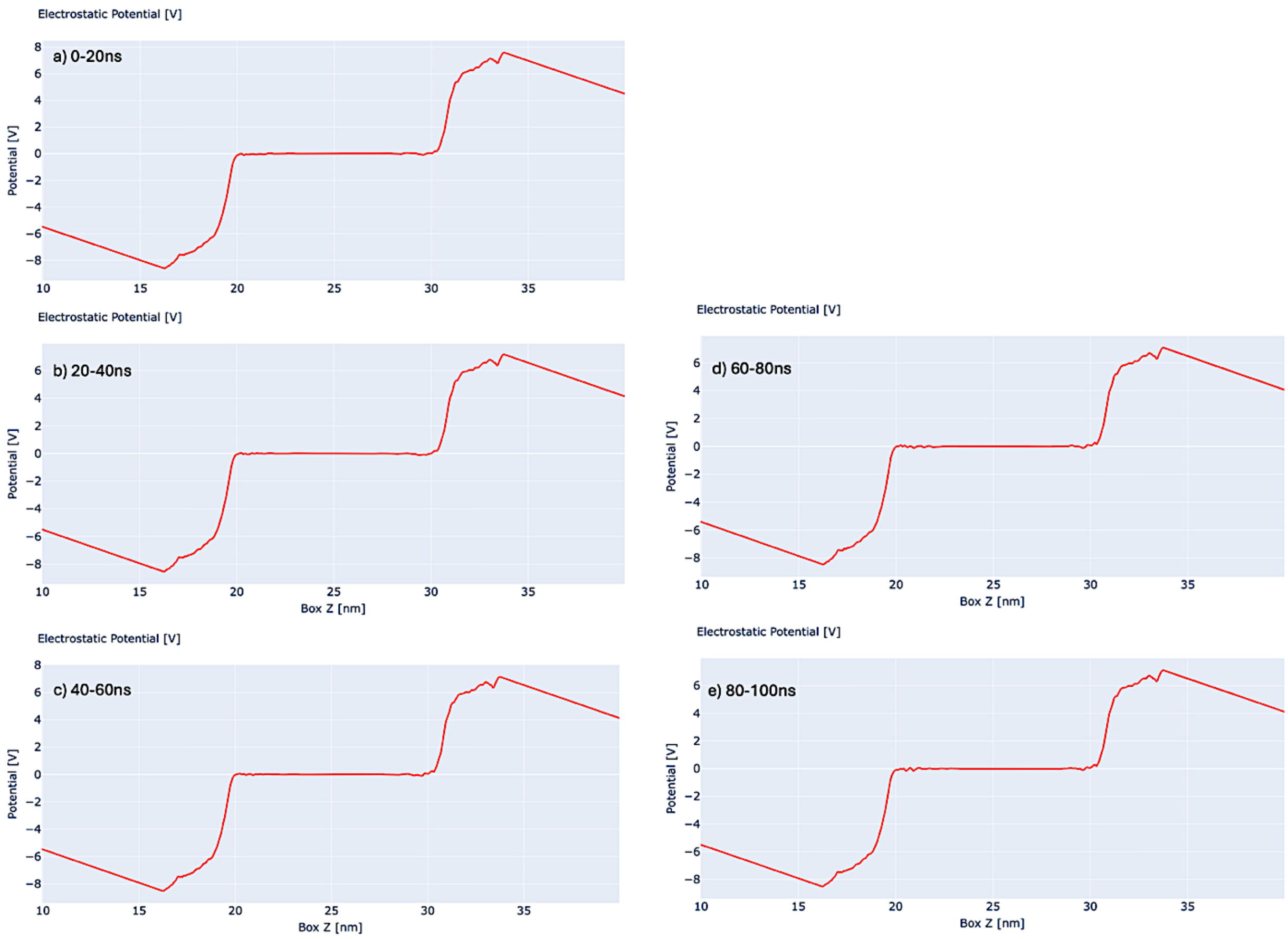

Figure S4: Block-resolved electrostatic potential profiles along the Z direction for the confined EFL$_4$FE/[PYR][TFSI]/RFL$_4$FR system, calculated independently over five consecutive 20 ns intervals of the 100 ns production trajectory: (a) 0–20 ns, (b) 20–40 ns, (c) 40–60 ns, (d) 60–80 ns, and (e) 80–100 ns. The profiles preserve the same overall shape, bulk-like central reference region, and interfacial potential features throughout the trajectory, supporting the temporal stability of the calculated electrostatic response.
